# Supplementary figures and images for: Providing personal information to the benefit of others
Source: PLoS One. 2020 Aug 19;15(8):e0237183. doi: 10.1371/journal.pone.0237183 (PMC7437809; doi:10.1371/journal.pone.0237183)

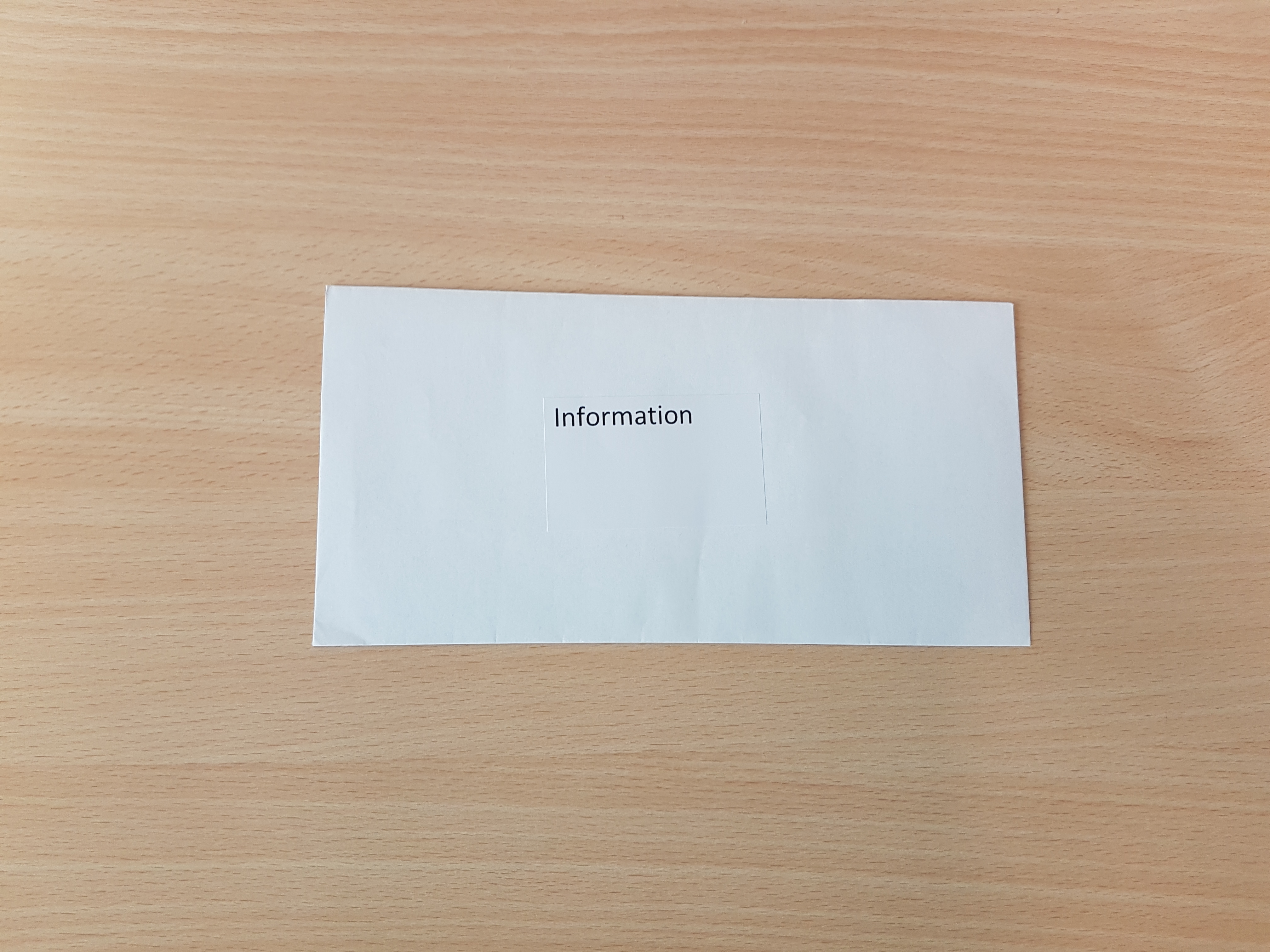

Supplement: S1 Fig — (TIF) [file pone.0237183.s001.tif]

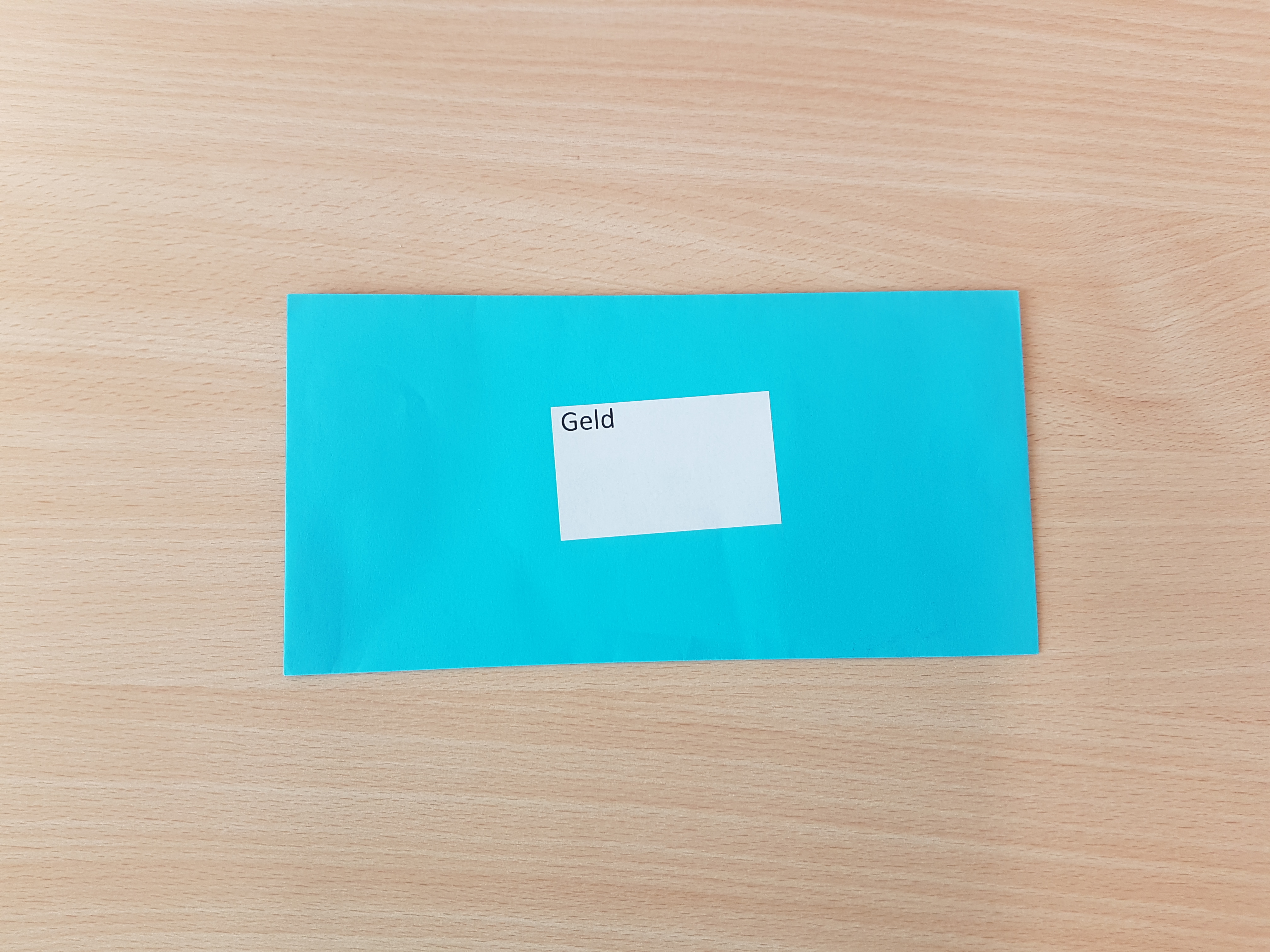

Supplement: S2 Fig — (TIF) [file pone.0237183.s002.tif]

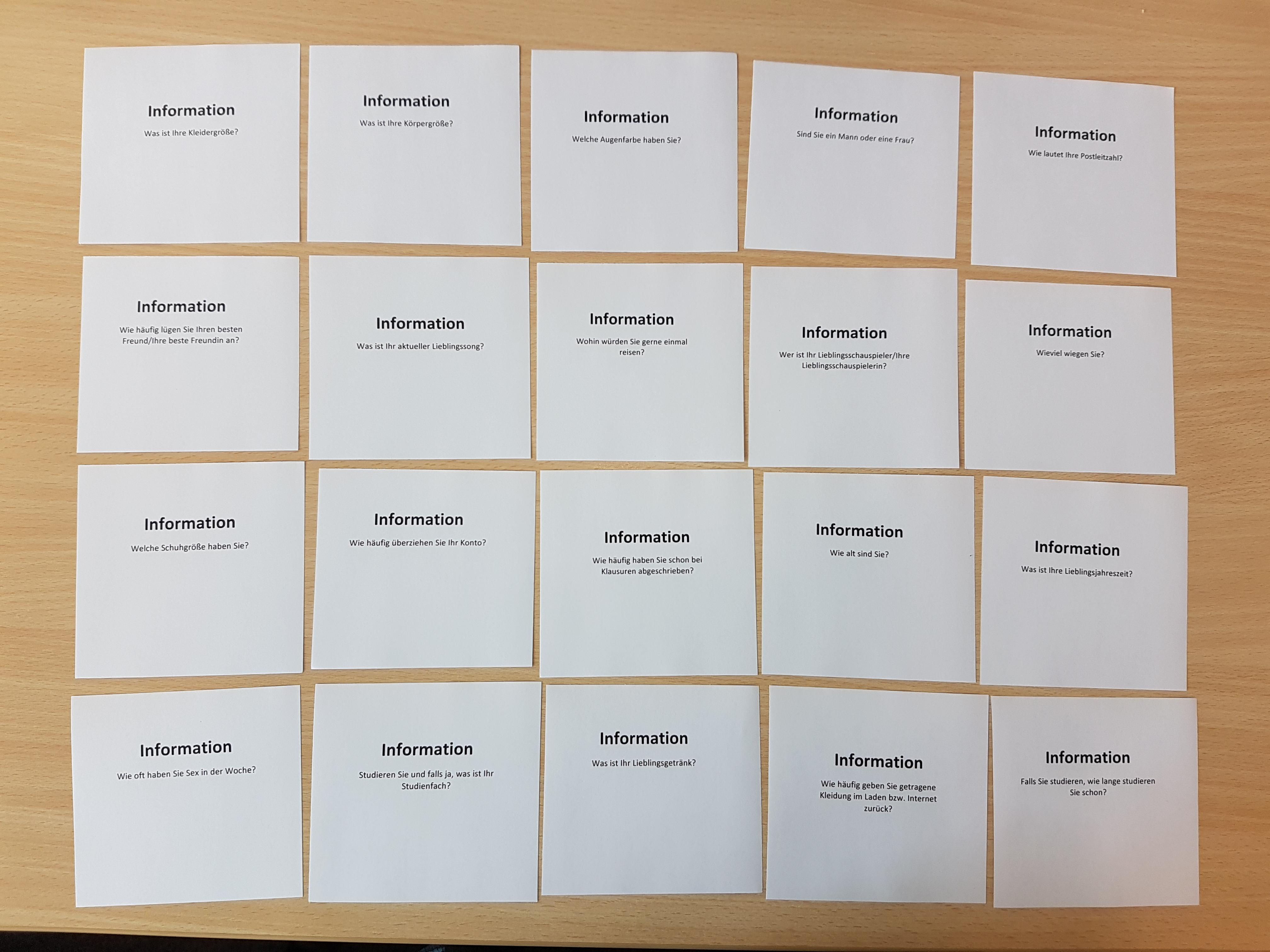

Supplement: S3 Fig — (TIF) [file pone.0237183.s003.tif]

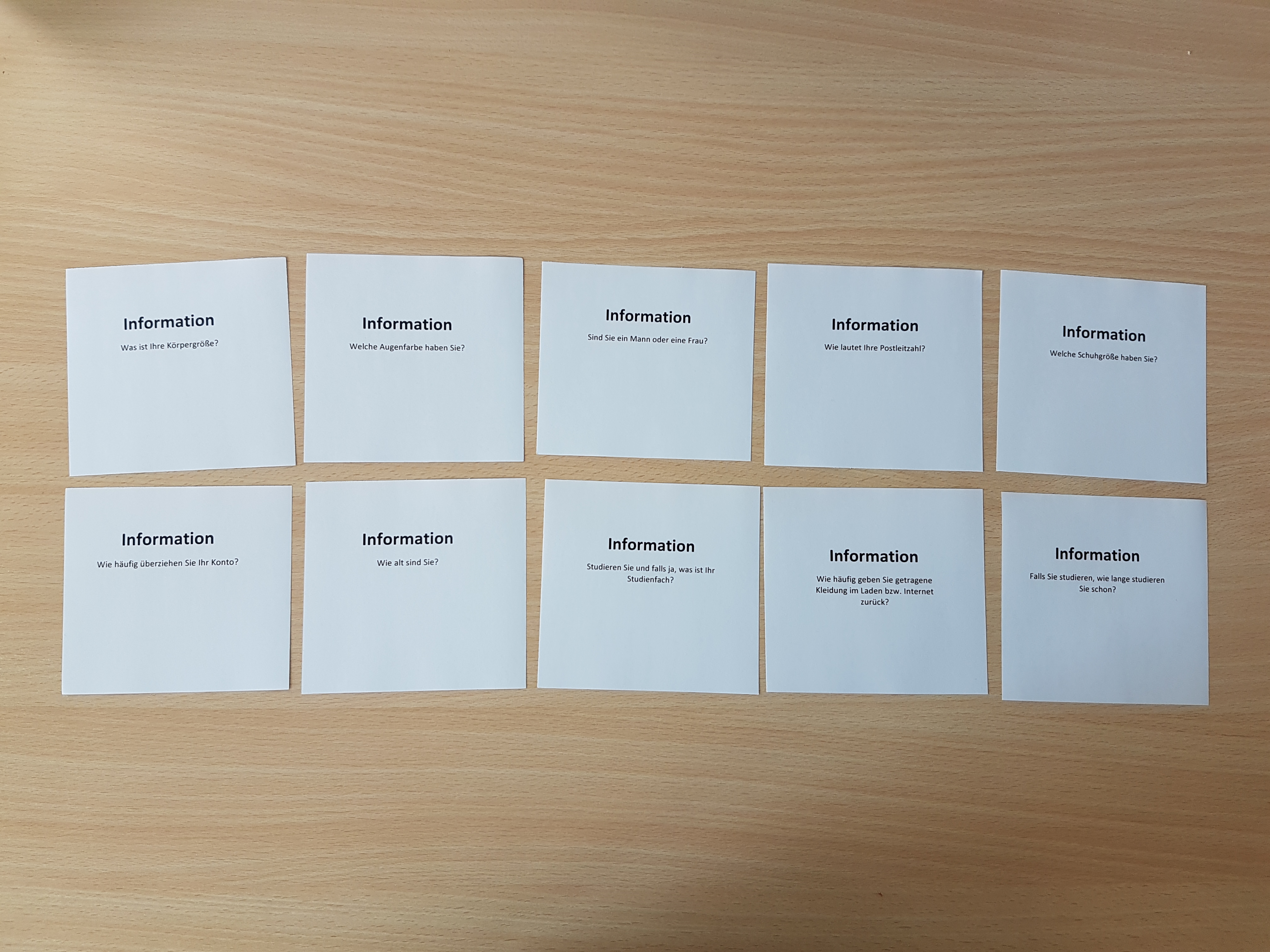

Supplement: S4 Fig — (TIF) [file pone.0237183.s004.tif]

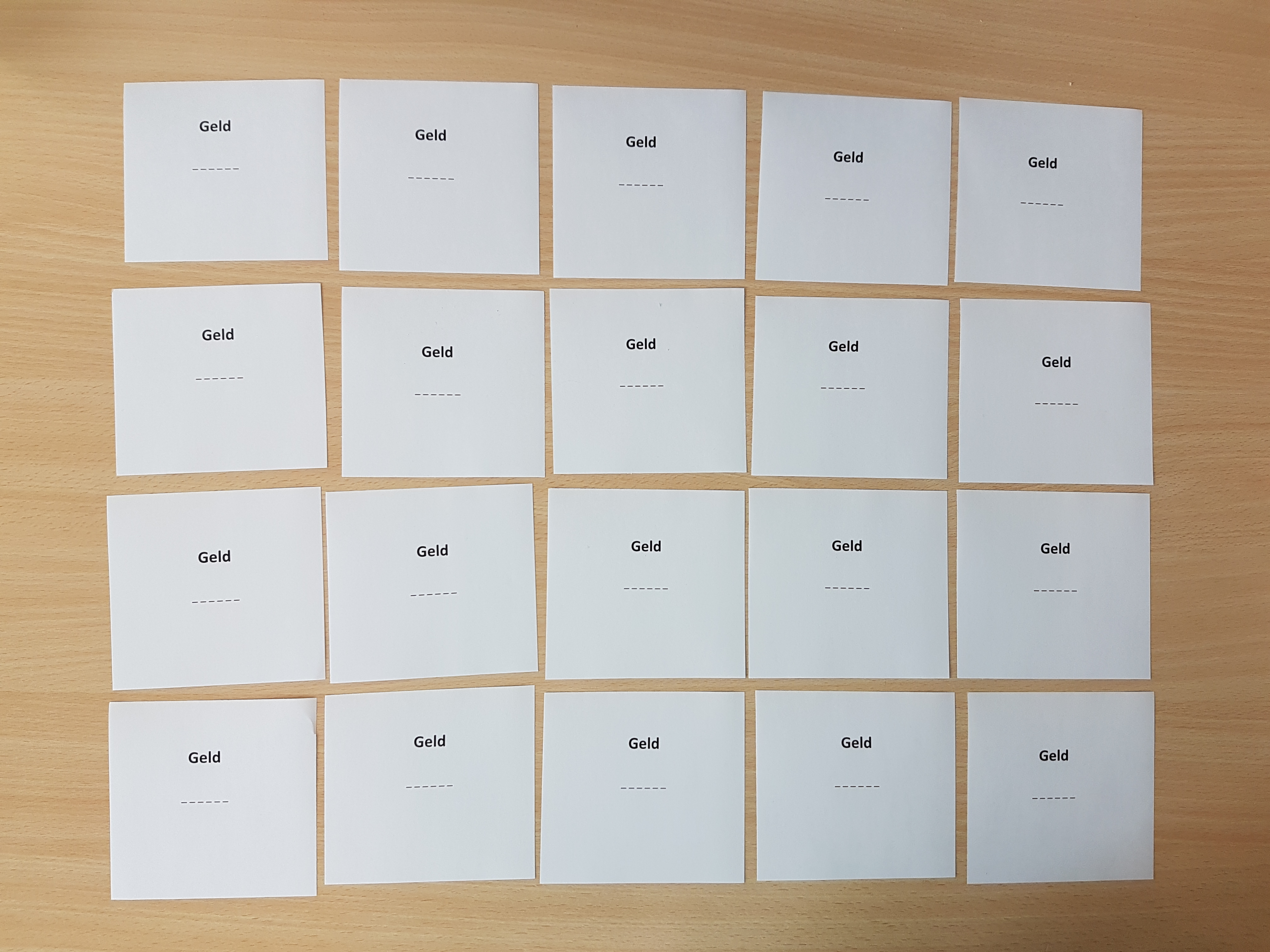

Supplement: S5 Fig — (TIF) [file pone.0237183.s005.tif]

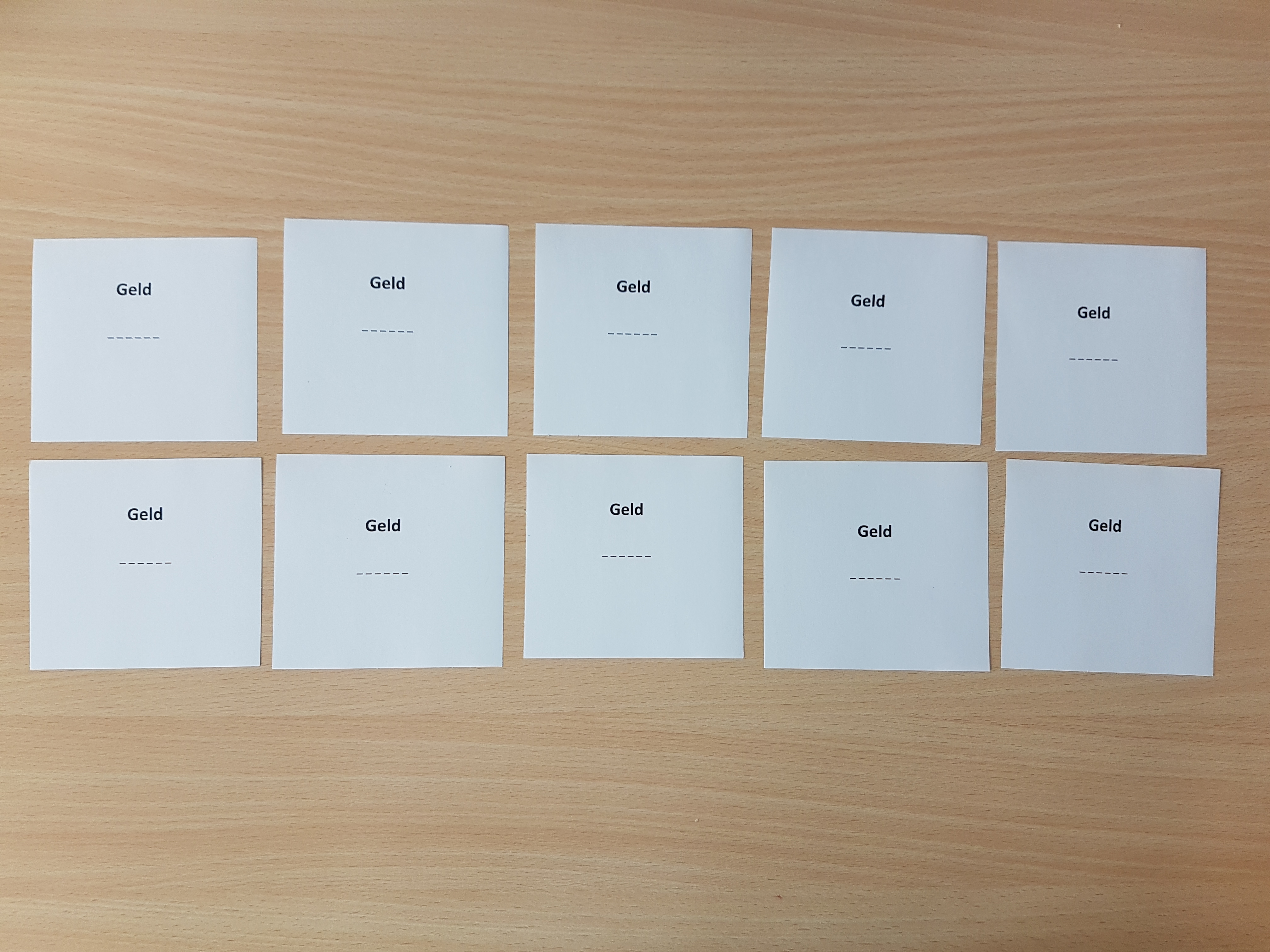

Supplement: S6 Fig — (TIF) [file pone.0237183.s006.tif]
