# Supplementary material for: Providing personal information to the benefit of others
Source: PLoS One. 2020 Aug 19;15(8):e0237183. doi: 10.1371/journal.pone.0237183 (PMC7437809; doi:10.1371/journal.pone.0237183)
Supplement: S1 File — (DOCX) [file pone.0237183.s007.docx]

S1 Questionnaire of the survey study (translated from German)

| Dear students,  female  male  **in this questionnaire, we would like to ask you** to evaluate 20 questions for a research project at the *Chair of Behavioral Economics*. For this purpose, please read each question carefully and then indicate how you evaluate the question.  years  Are you male or female?  How old are you?  What is your subject of study?  Thank you very much for your participation!  **very comfortable**  **very uncomfortable**  I think this question is…  **easy to answer**  **difficult to answer**  Answering this question, I feel…  What is your size?  [*questionnaire contains all 20 items as given in Table 3*] |
| --- |
