# Supplementary material for: Providing personal information to the benefit of others
Source: PLoS One. 2020 Aug 19;15(8):e0237183. doi: 10.1371/journal.pone.0237183 (PMC7437809; doi:10.1371/journal.pone.0237183)
Supplement: S2 File — (DOCX) [file pone.0237183.s008.docx]

S2 Instructions (translated from German)

S2.1 Instructions in the info condition

The translated instructions belong to treatment INFO. All deviations in treatment INFO_NC, INFO_RED, and INFO_RED_NC are given in square brackets.

| **Instructions for the experiment**  **General information**  We welcome you to this economic experiment. It is very important that you read the following instructions carefully. If you have questions, please get in touch with us.  Depending on your own and the other participants’ decisions, you can earn money in this experiment.  During the experiment, you are not allowed to talk to other participants of the experiment. Non-compliance with this rule leads to exclusion from the experiment and all payments. All decisions are made anonymously, i.e., none of the other participants gets to know the identity of a person who makes a certain decision. All payments are made anonymously, too, i.e., no participant gets to know the payoff of the other participants.  At the end of the experiment, you receive 2.50 Euro for showing up. During the experiment, you can earn additional money. On the following pages we explain to you the exact course of the experiment.  **Groups and rounds**   - You are part of a group with a total of **4 members.** - The experiment consists of **only one round.**   **Course of the experiment**   - You receive **20 information sheets** [*INFO_RED/INFO_RED_NC:* ***10*** ***information sheets*]** in a **white envelope** labeled with the word “Information”. Each of the information sheets contains a question about you. Take out all 20 information sheets [*INFO_RED/INFO_RED_NC: 10 information sheets*] of the white envelope, and decide for each of the information sheets whether you want to **provide the information about you for the group or keep it for yourself:**   - If you provide your information for the group, the **whole group profits** from this. For this purpose, please write the **truthful** answer to the question on the information sheet. Please put the answered information sheet back into the white envelope. For each answered information sheet which you put into the white envelope, **each group member (including yourself) receives 0.12 Euro** [*INFO_RED:* ***0.24 Euro***]**.**   [*INFO_NC: For each answered information sheet which you put into the white envelope, you* ***yourself receive 0.30 Euro, and each of the other three group members receives 0.12 Euro****.]*  [*INFO_RED_NC: For each answered information sheet which you put into the white envelope, you* ***yourself receive 0.60 Euro, and each of the other three group members receives 0.24 Euro****.]*   - - If you keep your information to yourself, **only you profit from this.** For this purpose, please leave the information sheet completely empty. Please put the empty information sheet back into the white envelope. For each empty information sheet you put into the white envelope, **you (but none of the other group members) receive 0.30 Euro** [*INFO_RED/INFO_RED_NC:* **0.60 Euro**]**.** - Your **payoff** is calculated as follows:  \| 0.12 Euro  [*INFO_RED/ INFO_RED_NC: 0.24 Euro*] \| times \| the number of information sheets which you and the other member of your group have provided for the group  [*INFO_NC/INFO_RED_NC: the number of information sheets which have been provided by the* ***other*** *members of your group*] \| \| --- \| --- \| --- \| \| + \|  \|  \| \| 0.30 Euro  [*INFO_RED/ INFO_RED_NC: 0.60 Euro*] \| times \| the number of information sheets you have kept for yourself  [*INFO_NC: 20* (*=number of information sheets in your envelope*)]  [*INFO_RED_NC: 10* (*=number of information sheets in your envelope*)] \| \| = \|  \| Your payoff \|  - After all participants have made their decisions, **all 20 information sheets** [*INFO_RED/INFO_RED_NC:* ***10 information sheets***] **should be put back into the white envelope**. - No participant of the experiment gets to know how many and which information sheets the other participants have provided and how many they have kept for themselves. Each participant only learns **her own payoff** after the experiment. - As soon as all participants have made their decisions the lab team collects the white envelopes. - Then, the lab team hands out a **questionnaire**. Please fill in the questionnaire while the lab team calculates your payoffs. After you have filled in the questionnaire, please stay at your cabin until we separately call you for payment.   **Data protection**   - **Please note:** The information about the group members which has been put into the white envelopes will be statistically evaluated anonymously, and destroyed by the experimenter immediately thereafter. At no time will information be assigned to the person of a single participant. Information will not be passed to third parties. Information will not be used for any other than research purpose, especially not for any direct or indirect commercial purpose.   **Good luck and thank you very much for your participation!** |
| --- | --- | --- | --- | --- | --- | --- | --- | --- | --- | --- | --- | --- |

S2.2 Instructions in the money condition

The translated instructions belong to treatment MONEY. All deviations in treatment MONEY_NC, MONEY_RED, and MONEY_RED_NC are given in square brackets.

| **Instructions for the experiment**  **General information**  We welcome you to this economic experiment. It is very important that you read the following instructions carefully. If you have questions, please get in touch with us.  Depending on your own and the other participants’ decisions, you can earn money in this experiment.  During the experiment, you are not allowed to talk to other participants of the experiment. Non-compliance with this rule leads to exclusion from the experiment and all payments. All decisions are made anonymously, i.e., none of the other participants gets to know the identity of a person who makes a certain decision. All payments are made anonymously, too, i.e., no participant gets to know the payoff of the other participants.  At the end of the experiment, you receive 2.50 Euro for showing up. During the experiment, you can earn additional money. On the following pages we explain to you the exact course of the experiment.  **Groups and rounds**   - You are part of a group with a total of **4 members.** - The experiment consists of **only one round.**   **Course of the experiment**   - You receive **20 money sheets** [*MONEY_RED/ MONEY _RED_NC:* ***10*** ***money sheets*]** in a **blue envelope** labeled with the word “Money”. Take out all 20 money sheets [*MONEY _RED/MONEY _RED_NC: 10 money sheets*] of the blue envelope, and decide for each of the money sheets whether you want to **provide the money for the group or keep it for yourself:**   - If you provide money for the group, the **whole group profits** from this. For this purpose, please write the word “GROUP” on the money sheet. Please put the labeled money sheet back into the blue envelope. For each labeled money sheet which you put into the blue envelope, **each group member (including yourself) receives 0.12 Euro** [*MONEY_RED:* ***0.24 Euro***]**.**   [*MONEY_NC: For each labeled money sheet which you put into the blue envelope, you* ***yourself receive 0.30 Euro, and each of the other three group members receives 0.12 Euro****.]*  [*MONEY_RED_NC: For each labeled money sheet which you put into the blue envelope, you* ***yourself receive 0.60 Euro, and each of the other three group members receives 0.24 Euro****.]*   - - If you keep your money to yourself, **only you profit from this.** For this purpose, please leave the money sheet completely empty. Please put the empty money sheet back into the blue envelope. For each empty money sheet you put into the blue envelope, **you (but none of the other group members) receive 0.30 Euro** [*MONEY_RED/MONEY_RED_NC:* **0.60 Euro**]**.** - Your **payoff** is calculated as follows:  \| 0.12 Euro  [*MONEY_RED/ MONEY_RED_NC: 0.24 Euro*] \| times \| the number of money sheets which you and the other member of your group have provided for the group  [*MONEY_NC/MONEY_RED_NC: the number of money sheets which have been provided by the* ***other*** *members of your group*] \| \| --- \| --- \| --- \| \| + \|  \|  \| \| 0.30 Euro  [*MONEY_RED/ MONEY_RED_NC: 0.60 Euro*] \| times \| the number of money sheets you have kept for yourself  [*MONEY_NC: 20* (*=number of money sheets in your envelope*)]  [*MONEY_RED_NC: 10* (*=number of money sheets in your envelope*)] \| \| = \|  \| Your payoff \|  - After all participants have made their decisions, **all 20 money sheets** [*MONEY_RED/MONEY_RED_NC:* ***10 money sheets***] **should be put back into the blue envelope**. - No participant of the experiment gets to know how many money sheets the other participants have provided and how many they have kept for themselves. Each participant only learns **her own payoff** after the experiment. - As soon as all participants have made their decisions the lab team collects the blue envelopes. - Then, the lab team hands out a **questionnaire**. Please fill in the questionnaire while the lab team calculates your payoffs. After you have filled in the questionnaire, please stay at your cabin until we separately call you for payment.     **Good luck and thank you very much for your participation!** |
| --- | --- | --- | --- | --- | --- | --- | --- | --- | --- | --- | --- | --- |
