# Supplementary material for: Providing personal information to the benefit of others
Source: PLoS One. 2020 Aug 19;15(8):e0237183. doi: 10.1371/journal.pone.0237183 (PMC7437809; doi:10.1371/journal.pone.0237183)
Supplement: S3 File — (DOCX) [file pone.0237183.s009.docx]

S3 Post-experimental Questionnaire (translated from German)

The translated questionnaire belongs to treatment INFO.

| **Questionnaire**  While we calculate your payoffs, we would like to ask you to fill in this questionnaire.  1. In your opinion, how many information sheets have the other members of your group provided for the group on average?  2. What was the reason for your personal provision decision in the experiment? Please explain.  3. If you have purchased something that meets your expectations, how often are you writing a customer rating in the internet?  Never  Very often  4. If you have purchased something that does not meet your expectations, how often are you writing a customer rating in the internet?  Never  Very often  5. If you consider purchasing something, how often do you read customer ratings in the internet?  Never  Very often  6. How helpful do you think are customer ratings in the Internet?  Helpful  Not helpful  7. How often do you post content in internet forums?  Never  Very often  8. Are you an active member of an internet forum?  Yes  No  9. If you have a question with regard to a specific issue, how often do you search for answers in internet forums?  Never  Very often  10. How helpful do you think that content in internet forums is?  Helpful  Not helpful  11. Do you donate for charitable purposes?  Yes  No  12. How important do you think it is to donate for charitable purposes?  Important  Not important  13. Do you engage in volunteer social projects?  Yes  No  14. How important do you think it is to engage in volunteer social projects?  Important  Not important  15. Are you male or female?  Female  Male  16. How old are you?  years  17. If you study, what is your subject of study?  Thank you! |
| --- |
|  |
