# Supplementary material for: Providing personal information to the benefit of others
Source: PLoS One. 2020 Aug 19;15(8):e0237183. doi: 10.1371/journal.pone.0237183 (PMC7437809; doi:10.1371/journal.pone.0237183)
Supplement: Attachment — Submitted filename: Response to Reviewers.docx [file pone.0237183.s010.docx]

Dear Reviewers,

Thank you very much for your assessment of our paper and the insightful reviews. We are very happy to submit a revised manuscript that addresses each point raised in the reviews. We very much hope that the revised manuscript now meets PLOS ONE’s publication criteria.

In this rebuttal letter, we address each point raised and describe in detail how we improved our paper based on this point.

Best regards

Bettina Rockenbach on behalf of all authors

**Reviewer #1: Providing personal information to the benefit of others
Referee Report**

***************
*** Summary ***
***************
The authors report the results of two public goods games. The first one is the standard version of the game, in which four players must decide how many of their 20 units of endowment they want to contribute to a common fund, granting higher earnings to the group, and how many they want to keep for themselves. The novelty in this design is a treatment in which participants do not contribute monetary tokens, but rather write down and contribute personal information details (20 different questions), varying in cognitive costs (associated to recall), and in emotional costs. In the MONEY and INFO treatments, there is also an exogenous variation in the cost of contribution (POSITIVE vs ZERO cost). The authors find lower provision in the INFO game compared to the standard MONEY game. They also find that implicit costs (cognitive and emotional) of specific information are correlated with the willingness to provide that specific information.

***********************
*** General comments ***
***********************
What I like the most about the paper is the contribution on bringing the “cooperation to a public good” component to the important debate on the disclosure and handling of personal information. The authors are aware of the relevant literature, and it helps a lot the reader to understand the aimed contribution of this study. The authors also do a good job in highlighting the advantages of bringing this problem to the lab (provided information is secured, and there should be lower trust concerns on how information will be handled).
My main concern is the connection between the very interesting examples provided in the abstract and in the introduction (traffic flows and health issues), and the type of requested information in the experiment. I understand that, as an experimentalist, sometimes one needs to sacrifice some external validity to gain control and internal validity. However, in the proposed design, I am afraid that the type of requested information, and the process to collect it, does not necessarily evoke the nature of “public good” that the disclosed information might have.
This might be problematic for two reasons. First, if the reason why my private information contributes to a greater knowledge is not clear, I have less incentives to truthfully report. In other words, I can contribute to the group, even providing false information (which mechanically can also happen in your game).

>> *Thank you for bringing up this relevant point. We designed our study to investigate whether there is a fundamental difference between ‘money’ and ‘information’ as units of provision to a public good. Due to the more foundational character of our study we abstracted from any specific application. And, importantly, to draw the parallel to money provision, the public good is not created by the aggregation of the specific information provided, but by the willingness to provide information.* *Sharing information (as providing money) creates a monetary benefit for others and this benefit does not depend whether or not the information is truthful, but only whether or not people are willing to share. This maps real world applications where truthful reporting is not a critical point, because the info is gathered automatically, e.g., location data or search tracing. Here people make the decision whether or not give consent to collect personal information and the truthful info is then conveyed. We clarified this point in lines 121-129 of the revised manuscript.*

Second, in the same way that you model an additional cost Gamma capturing implicit individual costs, one could think of an additional parameter (let’s call it Phi) that might capture other non-monetary costs (or benefits) of the aggregated information. The disclosed or “shared” information might trigger other type of behaviors in which I compare my information with the rest of the group (e.g., conformity). This concern does not invalidate the experiment. But I would like to see a discussion on this issue.

*>> This is an interesting suggestion, yet in our experiment participants receive no feedback on the aggregated information to avoid any confounds from psychological effects reaching beyond the mere provision of personal information. Therefore, they cannot gain additional benefits or costs from the aggregated information.* *To make info and money provision comparable, our experiment only uses one outcome dimension, which is money. We clarified this point in lines 161-164 of the revised manuscript.*

*************************************
*** Other concerns/questions/comments ***
*************************************
1. What do “social preferences” mean in this context? It is not clear if in the context of information disclosure, social preferences include more than the usual altruism and reciprocity concerns. For instance, shame or (dis-)conformism. In the same line, it is not clear what would be here to “maximize joint utility.”

>> *Thanks for addressing this point. As mentioned above, participants* *receive no feedback on the aggregated info and thus cannot gain additional benefits or costs from aggregated info. Nonetheless, we clarified that our parameter gamma may integrate various psychological costs (see line 158 of the revised manuscript) and rephrased the term ‘joint utility’ in ‘sum of all group players’ utilities’ (see line 198 of the revised manuscript).*

2. On the decision of making provision cost zero I do not think this is necessarily a bad decision, but as reader I would like to understand better why implement a cost zero, rather than a very small cost (e.g., €0.01).

*>>* *We decided to implement zero costs to investigate whether or not there is a difference between money and information provision under this boundary condition of true monetary indifference. Implementing a tiny cost would leave the question of what happens at the boundary unanswered. We clarified this point in lines 229-231 of the revised manuscript.*

*With zero costs, the game is a public goods game, but theoretically it no longer constitutes a social dilemma. In MONEY_NC average provision is 19.29 and not significantly different from full provision, hence we are faced with a situation where there is apparently no social dilemma. In INFO_NC, however, average provision is 17.8 and significantly different from full provision (p=0.0000), indicating that in the information condition even in absence of monetary provision costs, subjects still face a social dilemma which can only be ascribed to non-monetary provision costs.*

3. On the recruitment

Participants of this experiment had previous experience in this Lab? Experienced participants might be more aware of protocols for handling information, and therefore they might face lower psychological costs of providing information. I know that this feature will be balanced across treatments, but it might be useful as a control variable in case you have a combination of new and experienced lab participants.

*>> Unfortunately, this information was not collected in the experiment, but we take it as an excellent suggestion to collect this information in future experiments.*

4. On the confounding between cognitive and emotional costs
A different way to conduct the experiment would have been to ask all the participants to write their responses to the 20 questions, and then “destroy/tear/hide” those papers with information they were not willing to reveal. The main advantage would have been that cognitive costs associated to information recalling would have not played a role, and it would have been possible to focus on emotional costs. Moreover, I think (but you may disagree) that it would also allow to reduce misreport of information. I would like to know whether you considered a design with these features, but you might have discarded for some other reasons that I am not seeing (e.g., ethical or methodological issues).

*>> Thanks for bringing this up. If subjects are forced to answer all questions and then decide whether or not to provide the information, they may already carry some costs for answering each of the questions. They have to carry the cognitive costs, since they have to make up their minds about the cognitively more demanding questions. Additionally, they also incur (at least parts of) the emotional costs. Take, for example, the emotionally loaded question of ‘What is your weight?’. We know that people often avoid being exposed to this question by not at all measuring their weight, so it seems plausible that, if they are forced to write down the answer, they already incur emotional costs. If individual (cognitive and emotional) costs are already incurred by answering all questions, the decision of how many answers to provide reduces to a question of how social-minded a subject is. Our research focus is on the potential difference between ‘money’ and ‘information’ provision due to the potentially different cost structures. Therefore, we decided for an implementation that allows to forego costs by not providing the information.*

*********************
*** Minor comments ***
• The literature review is too detailed, you could shorten this section.

*>> We considerably shortened and condensed literature review.*

• Lines 314-316: Please provide a similar line for the comparison INFO vs. MONEY

*>> We included the comparison in lines 292-294 of the revised manuscript.*

• Lines 323-324: This sentence is hard to follow. Please rewrite.

*>> We rewrote the sentence, now in lines 301-304.*

**Reviewer #2: Review report for manuscript “Providing Personal Information to the Benefit of Others “**

This paper reports on a laboratory experiment that investigates contribution behavior based on personal information. The experimental design also involves treatments based on standard monetary incentives which makes comparison of two cases possible. The results indicate that contribution decisions in personal information treatments are related to emotional and cognitive costs; suggesting a conflict between standard public good behavior and information-based public goods.

This has not been one of the easiest reviews I have done. On the one hand, the research question is very relevant especially nowadays (with Covid-19 and also digitalization). The manuscript is decently-written and the experiment was well conducted. On the other hand, I found the experimental design too-disconnected from the real world and therefore artificial. Laboratory is ideal for investigating some specific research questions, but for a research question like this I do not think it is sufficient. It is true that the experiment is interesting, but a standard lab experiment does not seem enough to be enough to provide a strong evidence. Today, there are countless options to ask public opinion on a topic like this (online platforms, household surveys etc.)- and I am surprised the authors did not try to extend their study. In a nutshell, the study suffers from external validity problem strongly in my humble opinion.

>> *Thank you for addressing this important point. We designed our study to investigate whether there is a fundamental difference between ‘money’ and ‘information’ as units of provision to a public good. Due to the more foundational character of our study we abstracted from any specific application. To understand the mechanisms underlying differences in behaviour, lab experiments are valuable because they allow to control, e.g., the different dimensions of our questions. We designed our study as a laboratory baseline for future field studies which address more specific and topic related questions. Addressing these topics in the field adds external validity, but may also bring limitations due to legal concerns or the fact that apps are not available in all dimensions of interest. We clarified this point in lines 121-129 of the revised manuscript.*

Some specific comments:

1- The personal information shared by the participants/survey respondents are not even similar to those individuals consent in real life. Most importantly, individuals’ aversion to share personal information in the real world is strongly connected to reliability of institutions and decision makers’ trust levels in institutions. The conceptual framework simplifies the mechanism too much. Furthermore, when individuals give consent on using their personal information (such as internet browsing or health history), they cannot lie about their personal data. Because most of the time, algorithms and technologies automatically retrieve the information. Or if it is a public institution that collects the data, individuals refrain from lying. In the experiment, the instructions are asking the respondents to be honest, which does not seem to be enough.

*>> As mentioned above,* *due to the more fundamental nature of our study, our questions were not meant to mirror a specific real-life application, but selected to be informative for certain characteristics of questions (cognitive and emotional). Nonetheless, some information is very close to what is gathered in real-life applications, like the ‘objective’ questions (age, gender, zip-code) or the ‘preferences questions’ (favourite song, drink, movie, actor). Other questions may be retrieved more indirectly, e.g., BMI through size and height in online shopping. We clarified this point in lines 239-244 of the revised manuscript.*

*We perfectly agree with the reviewer that individuals’ propensity to share personal information is strongly connected to reliability of institutions and the decision makers’ trust in those institutions. In our experimental study, we fix this dimension between all conditions and show that even if there is trust in institutions, there is a difference between the provision of units money and information. Varying the trust in institutions may be a valuable extension in future research to investigate the optimal design of institutions depending on the voluntary provision of personal information.*

*To draw the parallel to money provision which is still the state-of-the-art in the laboratory study of public goods provision, the public good is not created by the aggregation of the specific information provided, but by the willingness to provide information. Sharing information (as providing money) creates a monetary benefit for others and this benefit does not depend on whether or not the information is truthful, but only on whether or not people are willing to share it in the first place. This maps real world applications where truthful reporting is not a critical point, because the info is gathered automatically, e.g., location data or search tracing. Here people make the decision whether or not give consent to collect personal information and the truthful info is then conveyed. We clarified this point in lines 121-129 of the revised manuscript.*

2- The paper can benefit from the literature on private-collective innovation. For example:

Gächter, S., von Krogh, G., & Haefliger, S. (2010). Initiating private-collective innovation: The fragility of knowledge sharing. Research Policy, 39(7), 893-906.

and studies of Eric von Hippel

Also the literature on experiments comparing decisions with monetary and non-monetary goods/bads. Some examples:

Davis, A. L., Miller, J. H., & Bhatia, S. (2018). Are preferences for allocating harm rational?. Decision, 5(4), 287.

Erkut, H. (2018). Social norms and preferences for generosity are domain dependent. WZB Discussion Paper.

Noussair, C.N. and Stoop, J., 2015. Time as a medium of reward in three social preference experiments. Experimental Economics, 18(3), pp.442-456.

Story, G. W., Vlaev, I., Metcalfe, R. D., Crockett, M. J., Kurth-Nelson, Z., Darzi, A., & Dolan, R. J. (2015). Social redistribution of pain and money. Scientific reports, 5, 15389.

*>> We are very grateful for pointing us to these strands of literature. We incorporated this into the discussion section.*

3- Implicit costs are not explained well. For example, cognitive cost is vaguely mentioned in page 3. But what does this correspond to in the real-world decision making? Why is it an important piece of the experimental design?

*>> When designing our experiment, we classified questions into different categories. Some being rather objective (e.g., shoe size or age), others being taste-based (e.g., favourite actor, or drink) and others being rather intimate (e.g., sex per week or cheating in exams). In our pre-test we identified that questions differ in their demand of cognitive and emotional resources and we classified the questions accordingly. These differences seemingly related to differing personal costs contribute to the implicit costs a participant faces when answering the question. Yet, we do not claim that these two types of costs are the only ones that matter. We see them as important representatives for psychological costs that may occur when providing information. We clarified this point in lines 239-244 of the revised manuscript by opening up for a broader scope of implicit costs of information provision.*

4- While the literature review section reviews the literature in detail (maybe even too detailed), the introduction cannot place the current study in the current literature and motivate the paper well. In other words, literature review and motivation should be better connected. As it is the literature review seems like a very separate section and the manuscript does not help reader to understand where this study aims to stand in the literature.

*>> We shortened and condensed literature review.*

5- Considering the facts that there are 4 members in each group, the total sample size is not too large and the experiment was not pre-registered, the reader gets curious about the power analysis.

*>> It is true that there are 4 members in each group, but note that the game is one-shot and the four members did not interact. Therefore, we have 52 to 56 independent observations in each of our treatments. In the power analysis we could not refer to a comparable experiment that would serve as a benchmark to estimate the effect size. We assumed an effect size of 0.20 and a power of 80% and thus expected to have sufficient power with a total of 200 participants. The Pseudo-R-squared in the regressions of Table 5 and Table 9 show that – ex post – the effect size of 0.20 is an appropriate, yet conservative, estimate.*
